# Triply Periodic Minimal Surface-Based Scaffolds for Bone Tissue Engineering: A Mechanical, *In Vitro* and *In Vivo* Study

**DOI:** 10.1089/ten.tea.2023.0033

**Published:** 2023-10-11

**Authors:** Ekaterina Maevskaia, Julien Guerrero, Chafik Ghayor, Indranil Bhattacharya, Franz E. Weber

**Affiliations:** ^1^ Center of Dental Medicine, Institute of Oral Biotechnology & Bioengineering, University of Zurich, Zurich, Switzerland.; ^2^ CABMM, Center for Applied Biotechnology and Molecular Medicine, University of Zurich, Zurich, Switzerland.

**Keywords:** osteoconduction, TPMS, 3D printing, bone substitute, microarchitecture

## Abstract

Triply periodic minimal surfaces (TPMSs) are found to be promising microarchitectures for bone substitutes owing to their low weight and superior mechanical characteristics. However, existing studies on their application are incomplete because they focus solely on biomechanical or *in vitro* aspects. Hardly any *in vivo* studies where different TPMS microarchitectures are compared have been reported. Therefore, we produced hydroxyapatite-based scaffolds with three types of TPMS microarchitectures, namely Diamond, Gyroid, and Primitive, and compared them with an established Lattice microarchitecture by mechanical testing, 3D-cell culture, and *in vivo* implantation. Common to all four microarchitectures was the minimal constriction of a sphere of 0.8 mm in diameter, which earlier was found superior in Lattice microarchitectures. Scanning by μCT revealed the precision and reproducibility of our printing method. The mechanical analysis showed significantly higher compression strength for Gyroid and Diamond samples compared with Primitive and Lattice. After *in vitro* culture with human bone marrow stromal cells in control or osteogenic medium, no differences between these microarchitectures were observed. However, from the TPMS microarchitectures, Diamond- and Gyroid-based scaffolds showed the highest bone ingrowth and bone-to-implant contact *in vivo*. Therefore, Diamond and Gyroid designs appear to be the most promising TPMS-type microarchitectures for scaffolds produced for bone tissue engineering and regenerative medicine.

**Impact Statement:**

Extensive bone defects require the application of bone grafts. To match the existing requirements, scaffolds based on triply periodic minimal surface (TPMS)–based microarchitectures could be used as bone substitutes. This work is dedicated to the investigation of mechanical and osteoconductive properties of TPMS-based scaffolds to determine the influencing factors on differences in their behavior and choose the most promising design to be used in bone tissue engineering.

## Introduction

Typically, bone tissue can regenerate by itself, but in demanding cases such as tumor resections or extended defects that pose the risk for non- or malunions, the use of bone grafts is mandatory.^1–3^ The application of autografts remains the gold standard in medicine,^4^ although it comes with serious drawbacks.^5^ The ideal bone substitute should provide biomechanical support with the creation of a suitable microenvironment for cells to adhere, proliferate, and differentiate,^6^ ultimately leading to bone ingrowth and creeping substitution, the main features of osteoconduction.^7^ To match these requirements on the level of the microarchitecture of the scaffold, the triply periodic minimal surface (TPMS) designs appear as an interesting option.

TPMSs are infinite surfaces with periodicity in three dimensions with no self-intersections and zero mean curvature.^8,9^ Applications of TMPSs are multidisciplinary,^10^ including satellites, aircrafts, and electric vehicles, and recently started to be tested for bone tissue engineering.^11^ The initial reason for this arising interest is that their tortuosity is similar to trabecular bone.^12^ Moreover, such continuous surfaces with smooth joints cause fewer stress concentrations and correspond to higher mechanical characteristics,^13^ lead to the enhancement of cell adhesion and proliferation,^14^ and thus to increased bone tissue ingrowth.^15^ The interconnectivity of pores is associated with rapid bone regeneration, vascularization, and material resorption.^16,17^ In addition, lightweight structures allow minimal material usage and therefore lower cost of the resulting scaffold and decreased time for its degradation *in vivo*.

Several TPMSs are known, however, Gyroid, Diamond, and Primitive are the most frequently tested for tissue-engineered bone substitutes. Gyroid scaffolds were shown to be more permeable compared with Primitive and Diamond scaffolds, which plays a role in cell seeding efficiency, cellular infiltration, differentiation, and new tissue formation *in vivo*.^18^ Diamond structures were shown to exhibit higher interconnectivity levels.^19^ Scaffolds should provide enough mechanical stability^20^ and match the mechanical properties of peripheral bone tissues to avoid stress shielding.^21^ The best performance in terms of strength, elasticity, and energy absorption was found for the Diamond design.^22^

In other studies, Primitive lattice microarchitectures had a higher elastic modulus than lattice microarchitectures based on Diamond or Gyroid.^23^ Graded Gyroid designs appeared superior to graded Diamond designs in terms of Young modulus, strength, ductility,^12^ and nongraded Gyroid designs showed a higher fatigue strength than strut-based structures.^24^ Taken together, a huge variety of small sets of TPMS-based scaffolds have been tested for limited aspects but the optimal choice of a TPMS microarchitecture for bone tissue engineering is still elusive.

There are also contradicting results on the influence of scaffold microarchitecture on cell growth. For example, Diamond scaffolds were shown to be stiffer than Gyroid and Primitive resulting in a higher cell death level^25^ and cell viability within Gyroid scaffolds was shown to be higher at all measured time points.^12^ However, another study indicated good cytocompatibility of Diamond structures.^26^ Alkaline phosphatase gene expression values were higher for Primitive microarchitecture,^19^ suggesting enhanced cell differentiation. Altogether, at present, the best TPMS structure for cell proliferation and/or differentiation is still unknown.

Regarding *in vivo* studies, the influence of TPMS-based designs on scaffold osteoconductive properties is even less investigated. There are only a few studies involving the implantation of TPMS-based scaffolds, and all of them were performed with a unique type of TPMS design. An early osteointegration ability,^27^ higher osteoconduction, and bone metabolic activity compared with cross-hatch, Lattice-like structures were shown for Primitive microarchitecture.^28^ Gyroid scaffolds were shown to provide bone tissue ingrowth after 8 weeks of implantation^29^ with better regenerative performance than with a Lattice structure.^30^ Titanium scaffolds based on Diamond designs showed efficient bone regeneration ability in femur sites but yielded poor results in skull defect sites.^26^ Nevertheless, to our knowledge, there are no comparative studies with scaffolds based on different types of TPMS microarchitectures. A lack of knowledge about mechanical and biological properties or structure–function relationships is not limited to TPMS microarchitectures but for the majority of 3D-printed scaffold architectures was stated in several review articles.^31,32^

Because TPMS structures are assumed to be promising microarchitectures for bone tissue engineering, existing results are still incomplete and contradictory. The goal of this study was to compare Diamond, Gyroid, and Primitive TPMS microarchitectures realized by the same methodology and material and to test them in terms of mechanics, *in vitro* cell seeding with human bone marrow stromal cells (hBMSCs), and *in vivo* implantation in the calvarial bone of rabbits in comparison with a standard Lattice structure.^33^

## Materials and Methods

### Scaffolds production and characterization

Three-dimensional models for scaffold production were designed with the use of nTopology v.3 software. The wall thickness was 0.2 mm for TPMS designs and 0.3 mm for the Lattice design. The minimal pore size or constriction of all microarchitectures was 0.8 mm. Based on the corresponding “.stl” files, scaffolds were 3D printed using CeraFab 7500 (Lithoz) with the photosensitive hydroxyapatite-based slurry LithaBone™ HA 400 (Lithoz) (Table 1). After cleaning of 3D-printed scaffolds, they were sintered at 1300°C. Owing to the high sintering temperature, no additional sterilization of the samples was needed.

**Table 1.** Illustration of the Scaffolds Used for Mechanical Testing, In Vitro 3D-Cell Culture or In Vivo Implantation Exemplified with the Diamond Microarchitecture

a) Mechanical testing	b) *In vitro* hBMSCs culture	c) *In vivo* implantation
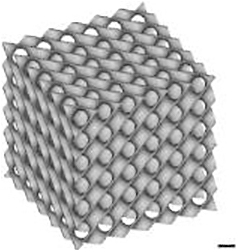	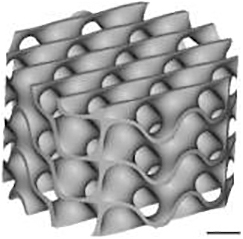	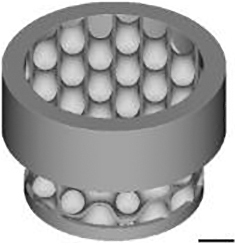

Scale bars of 1 mm are provided.

To measure the accuracy of the printing process, three scaffolds of each type were scanned using μCT SkyScan 1272 (Bruker) with the parameters listed in Table 2 (first scanning protocol). For the reconstruction the NRecon software was used; analysis of volume and surface area was made using CTAn software with the same thresholding for all scans. All software were provided by Bruker. Parameters such as porosity and ratio of surface area to volume were calculated and compared with the ones predicted by our 3D models.

**Table 2.** Parameters Used for μCT Scanning

Scanning protocol	Voltage	Current	Filter	Pixel size	Rotation step
Scaffold after 3D printing	90 kV	111 μA	Al 0.5 + Cu 0.038	10 μm	0.3°
Scaffold with tissue after implantation	80 kV	125 μA	Al 0.5 + Cu 0.038	10 μm	0.3°

The images obtained after μCT scanning were used to calculate the maximal sphere diameter by ImageJ (NIH) as the diameter of the largest circle that fits into the pore structure of the scaffold, as previously described.^33^ The transparency defined as a percentage of material-free area in the projection of the scaffold in the different spatial directions^34^ was calculated in three projections of the scaffolds (view from the top, the front, and the edge between the front and its side to the right; Table 4).

### Mechanical testing

For mechanical testing, the cubic models were designed with a side size of 7.8 mm [Table 1 (a)]. Mechanical properties of eight scaffolds per group were tested with the universal testing machine ROELL Z2.5 MA 18-1-3/7 (Zwick) by applying compressive load at a speed of 1 mm/min in the direction of building layers. The TestXpert V11.02 software (Zwick) was used to determine the breaking point and analyze the stress-strain curve. After reaching the breaking point of each scaffold, images were taken with the use of the digital microscope VHC 2000 (Keyence).

### Cell seeding of hBMSCs

Cylindrical scaffolds with a diameter of 6 mm and a height of 4 mm were produced for the cell seeding experiment [Table 1 (b)]. In this study, hBMSCs from three different donors were received from the Department of Biomedicine (University of Basel). In addition, two commercial cell lines of hBMSCs were used: No. SCC034 (Sigma-Aldrich) and No. PT-2501 (Lonza). All hBMSCs, both from donors and commercial cell lines, were used for the experiments with all types of microarchitectures.

The experiment included testing four different types of scaffolds with control and osteogenic medium over up to 21 days of culture. The control medium consisted of αMEM (No. 22571-020) with the addition of 10% fetal bovine serum (No. 26140-079), 1% HEPES (1 M) (No. 15630-056), 1% sodium pyruvate (100 mM) (No. 11360–070), and 1% of penicillin and streptomycin (No. 15140-122) with l-glutamine (No. 25030-024) (all reagents were from Gibco). The osteogenic medium was prepared at a final concentration of 50 μM of ascorbic acid (No. A8960), 10 mM of β-glycerophosphate (No. G9422), and 100 nM of dexamethasone (No. D2915) (Sigma-Aldrich). Single scaffolds were placed into single wells of 12-well plates, which were previously coated with a water solution of 2% agarose to prevent cell-plastic attachment.

The seeding procedure was performed as follows. First, 50 μL of control medium with the hBMSCs with a concentration of 2.6 × 10^4^/cm^2^ were added on the top of each scaffold. After 1 h of incubation at 37°C within the cell culture incubator to favor cell attachment, 2 mL of control medium were added. On the next day, the control medium was harvested from each seeded well for measurement of the cell seeding efficiency using CyQUANT™ Cell Proliferation Assay (No. C7026) (Thermo Fisher). Next, 2 mL of osteogenic or control medium were added to the well containing the previously seeded scaffold. Cells were cultured for 21 days under a 5% CO_2_ atmosphere at 37°C, with medium change twice a week.

Cell attachment and proliferation within scaffolds were observed under the CKX53 optical microscope (Olympus) on days 10 and 21. Before taking pictures, scaffolds were transferred into new wells to make sure that only cells within the scaffold were visible.

#### Metabolism study

The metabolic activity of hBMSCs was measured with the Alamar Blue assay. Resazurin sodium salt (No. R7017-1G) (Sigma-Aldrich) was diluted in PBS at a concentration of 50 μM and then mixed with a culture medium at a concentration of 10%. Cells were incubated in the solution for 4 h at 37°C within the cell culture incubator. Then, fluorescence was measured with the Synergy HT spectrophotometer (BioTec) at an excitation wavelength of 530 nm and an emission wavelength of 590 nm. Data were normalized to the seeding efficiency within each scaffold and the number of cells at day 0.

#### Differentiation

To study the differentiation of hBMSCs, the scaffolds were crushed and extracted after 10 and 21 days with the tissue lyser (QIAGEN) in the presence of QIAzol lysis reagent (QIAGEN). Then, RNA isolation was performed using RNeasy Plus Mini kit (No. 74034) (QIAGEN) and cDNA was obtained with the use of iScript cDNA Synthesis Kit (No. 1708891) (Bio-Rad) with the Mastercycler gradient (Eppendorf). Thereafter, cDNA was mixed with the iTaq Universal SYBR Green Supermix (No. 1725124) and the primers for the following genes (Bio-Rad): *SP7*, *ALPL*, *COL1A1*, *RUNX2*, *OPN*, *OCN*, *CAV1* (osteogenesis-related genes), *GAPDH*, and *18S* (reference genes). Real time quantitative polymerase chain reaction (RT-qPCR) was performed with the use of CFX Connect (Bio-Rad), followed by the determination of the relative gene expression by the 2^−ΔΔCt^ method. The data were normalized to the expression of both reference genes at day 0.

### Implantation

For *in vivo* implantations, scaffolds with a diameter of 6 mm and a height of 5 mm were produced [Table 1 (c)]. To prevent their falling inside the defect, the outer ring with a diameter of 7.5 mm and a height of 2.5 mm was added to the upper part and another ring with a thickness of 0.3 mm, an outer diameter of 6.0 mm, and a height of 0.5 mm was included into the bottom part of the scaffolds.

All animal procedures were approved by the Animal Ethics Committee of the local authorities (Canton Zurich, 065/2018 and 090/2021) and performed following the ethics criteria contained in the bylaws of the Institutional Animal Care and Use Committee. The procedure was carried out as described earlier.^35^ In brief, the scaffolds were implanted into calvarial defects of 12 rabbits (female, 26-week-old, New Zealand white rabbit). All four different types of scaffolds were implanted per animal and the overall pattern of the arrangement of the scaffolds was rotated clockwise by one position from one animal to the next. Four weeks after the implantation, the animals were anesthetized and sacrifized by an overdose of pentobarbital.

Next, μCT scanning of the scaffolds was performed using SkyScan 1272 (Bruker) with the parameters listed in Table 2 (second scanning protocol). Data were visualized using DataViewer and CTVox. Analysis of bone tissue ingrowth was made using CTAn with the same thresholding for all scans. The region of interest (ROI) for implanted scaffolds was defined as the inner part of the scaffold without the outer ring or the surrounding tissue. The volume of mineralized tissue was determined and normalized to the volume of ROI. For the bone-to-implant contact study, the volume of mineralized tissue was measured in the area close to the inner part of the scaffold's surface at a distance of three pixels (30 μm).

### Statistical analysis

All data were analyzed using GraphPad Prism v.5.01 (GraphPad Software). A normalization test was performed in each case and a parametric or nonparametric analysis was selected in accordance. For the comparison of mechanical characteristics, a one-way analysis of variance was used followed by Bonferroni's multiple comparison test. For the comparison of two independent groups, the Mann–Whitney test was used, and for several groups in other experiments, the Kruskal–Wallis test followed by Dunn's multiple comparison tests was applied. Jonckheere–Terpstra test was used to determine if there is a statistically significant decreasing level of constriction between each group. Results were considered significant with *p* < 0.05 and presented in graphs as a box-plot diagram with the median, 25th and 75th quartiles, and minimum and maximum values as whiskers.

## Results

### Scaffolds production

Scaffolds' parameters defined by the 3D models and measured by μCT scanning after printing are listed in Table 3. The produced samples closely matched the designed models in terms of volume, surface area, and porosity. The lowest porosity and surface-to-volume ratio were measured in the Primitive design with 70.1% ± 1.4% and 8.7% ± 0.1%, respectively. Three other structures showed closely related porosity, however, Diamond and Gyroid microarchitectures presented the highest surface-to-volume ratio with 19.1 ± 0.5 1/mm and 17.6 ± 0.3 1/mm, respectively, making them the most promising candidates for cell attachment. The lowest volume measured in scaffolds was observed with Diamond and Lattice designs with 12.0 ± 0.3 mm^3^ and 12.0 ± 1.2 mm^3^, respectively, corresponding to the least material consumption during the printing process.

**Table 3.** Comparison of Parameters Calculated with the 3D Models and Measured with μCT on the Printed Scaffolds

	Diamond 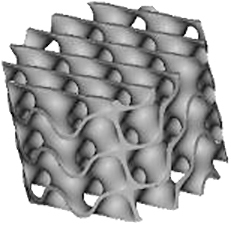		Gyroid 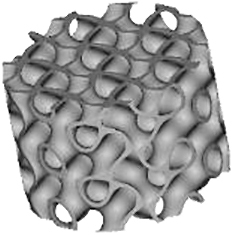		Primitive 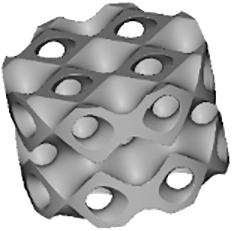		Lattice 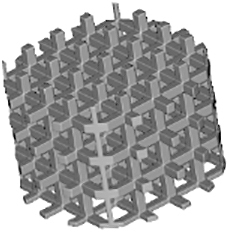	
	Model	μCT	Model	μCT	Model	μCT	Model	μCT
Volume (mm^3^)	12.7	12.0 ± 0.3	15.1	14.8 ± 0.2	20.6	19.5 ± 0.7	13.3	12.0 ± 1.2
Surface area (mm^2^)	224.2	228.6 ± 6.6	260.1	261.0 ± 5.3	171.7	169.2 ± 7.9	178.7	152.1 ± 15.3
Surface area/volume (1/mm)	17.7	19.1 ± 0.5	17.3	17.6 ± 0.3	8.3	8.7 ± 0.1	13.4	12.7 ± 0.6
Porosity (%)	82.1	81.7 ± 0.5	78.7	77.3 ± 0.3	70.8	70.1 ± 1.4	81.2	81.8 ± 1.9
Max sphere diameter (mm)	1.17 ± 0.02		0.81 ± 0.02		1.63 ± 0.17		0.79 ± 0.02	

Values are presented as mean ± standard deviation.

The diameter of the largest 2D sphere that fits into pores was determined by μCT images. Gyroid and Lattice structures yielded the lowest values with 0.81 ± 0.02 mm and 0.79 ± 0.02 mm, respectively, followed by Diamond design with 1.17 ± 0.02 mm. The highest value of 1.63 ± 0.17 mm was found for Primitive microarchitecture.

The Lattice scaffold occurred to be the most transparent in all three projections (Table 4). The transparency of the Diamond structure is limited to one spatial direction with 23%, although Gyroid was associated with the lowest values with 8% and 9%. Among the studied TPMS-based microarchitectures Primitive scaffold was the most transparent.

**Table 4.** Transparency of All Scaffolds in Three Spatial Directions

	Top 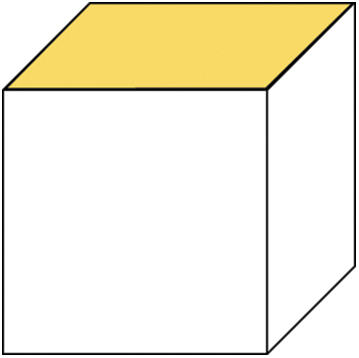	Front 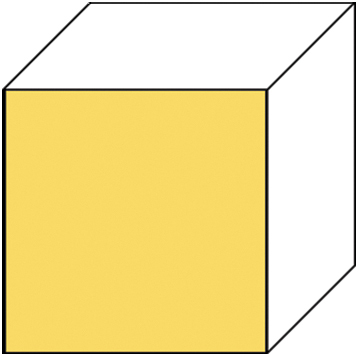	Edge 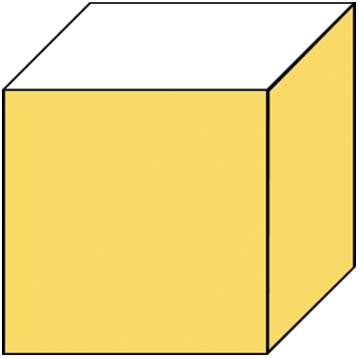
Diamond	0%	0%	23%
Gyroid	8%	9%	0%
Primitive	16%	11%	6%
Lattice	43%	42%	31%

### Mechanical characteristics

**FIG. 1.**
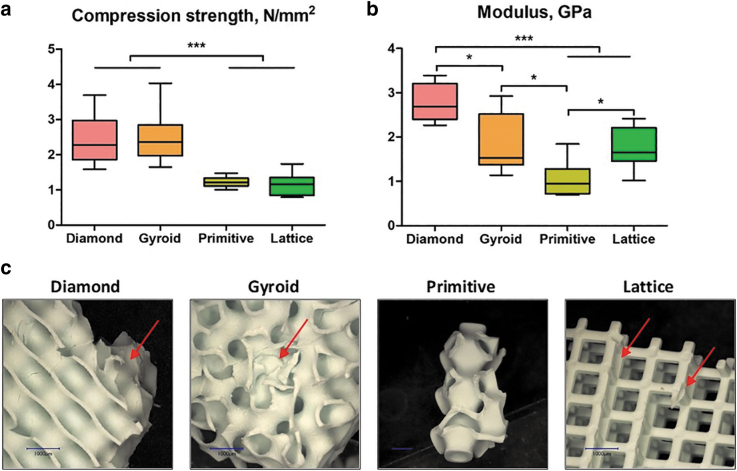
Mechanical testing of the scaffolds: compression strength **(a)** and elastic modulus **(b)**, *p < 0.05; ***p < 0.005. Illustration of scaffolds after reaching the breaking point **(c)**. *Arrows* indicate the breaking profile. Scale bars of 1 mm are provided.

The scaffold's compression strength and Young's modulus are listed in Figure 1. We observed that the compression strength of Diamond and Gyroid samples was two-fold higher compared with Primitive and Lattice and in the range of human cancellous bone (2–12 MPa).^36^ Concerning the elastic modulus, all tested samples occurred to meet trabecular bone values (0.2–5 GPa)^37^; however, they significantly varied from each other with Primitive microarchitecture having the lowest elasticity, 1.1 ± 0.4 GPa, whereas Diamond showed the highest one with 2.8 ± 0.4 GPa.

The breaking profile differed between the studied microarchitectures. In particular, the Lattice structure was usually broken at the interconnection of the layers of rods and disassembled in layers of grids (Fig. 1c). Diamond and Gyroid scaffolds remained beside smaller chip-offs largely intact, whereas for the Primitive microarchitecture only a small central piece was usually left intact and the rest was disassembled in small fragments (Fig. 1c).

### *In vitro* study

**FIG. 2.**
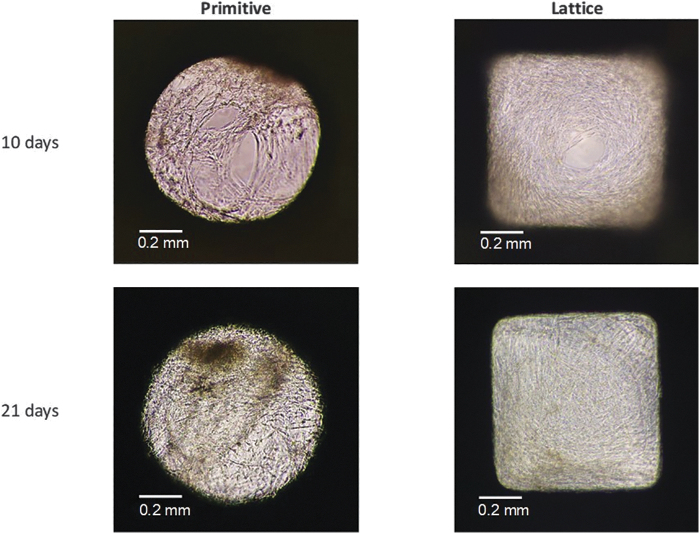
Growth of hBMSCs on Primitive and Lattice scaffolds showing the inside of the pores after 10 and 21 days. *Dark brown* spots correspond to mineralized areas. hBMSC, human bone marrow stromal cell.

In all conducted experiments, the seeding efficiency was ∼90% for scaffolds and monolayer culture groups (data not shown). Owing to the low transparency of Diamond and Gyroid scaffolds (Table 4), it was not possible to visualize hBMSCs growth inside these samples. Therefore, the distributions of hBMSCs only in Primitive and Lattice scaffolds are presented in Figure 2. We were able to observe that after 10 days hBMSCs were attached to the surface of our samples and proliferated. After 21 days, the pores were completely colonized by cells, and some nuclei of mineralization were visible for cells cultured in the osteogenic medium (Fig. 2, dark brown).

**FIG. 3.**
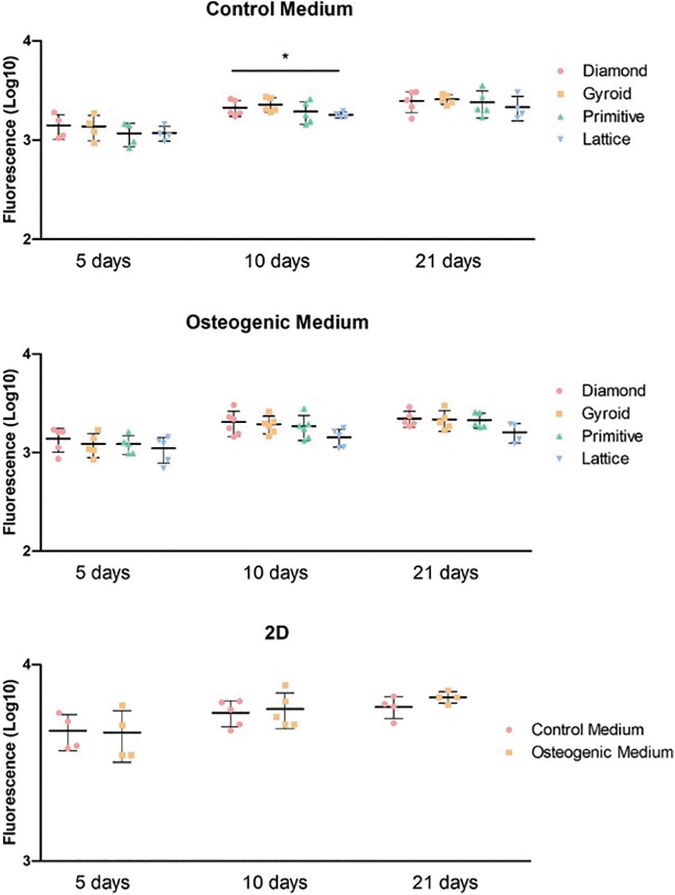
Cell metabolism in scaffolds with TPMS or lattice microarchitectures and 2D cultures at 5, 10, and 21 days. *p < 0.05. TPMS, triply periodic minimal surface.

We observed a significant increase in fluorescence as an equivalent of metabolism during the 21 days of culture in all conditions, confirmed by the Jonckheere–Terpstra test (Fig. 3). The scaffold type did not affect metabolism. The only statistical significance (*p* = 0.01) was found at 10 days in the control medium with the highest results for the Gyroid scaffold and the lowest ones for the Lattice. Concerning the osteogenic medium, the Lattice structure showed a lower level of fluorescence at all time points.

To study hBMSC differentiation, mRNA was extracted at different time points (10 and 21 days), and RT-qPCR was performed (Supplementary Fig. S1). The use of an osteogenic medium led to higher expression of bone-related genes in hBMSCs. The difference was more significant in the case of early markers of osteogenic differentiation (i.e., *ALPL*, *COL1A1*, and *RUNX2*). In contrast, for the late markers (i.e., *OPN*, *OCN*, *CAV1*), there were almost no differences between culture media. Besides, for longer culture periods (21 days), cells highly expressed most of the genes studied. Owing to donor variability, it was not possible to detect any influence of microarchitecture on hBMSC differentiation for any of the genes representing early or late markers.

### *In vivo* study

**FIG. 4.**
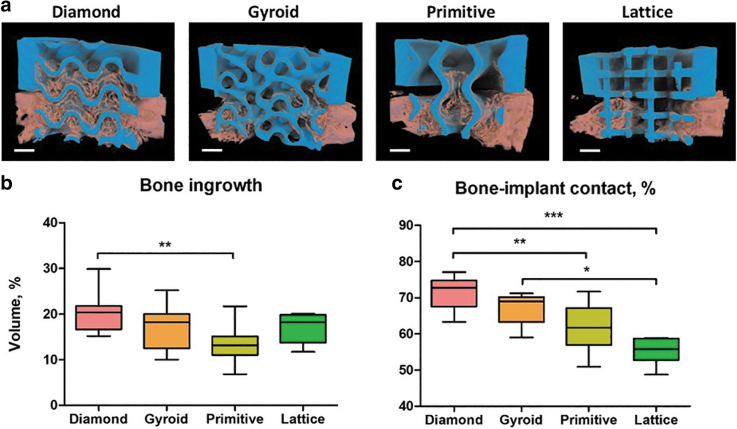
Bone regeneration in calvarial defects treated with scaffolds based on TPMS or lattice microarchitectures. Visualization of μCT scanning with CTVox. The scaffold appears in *blue* and the mineralized tissue in *pink*, scale bars of 1 mm are provided **(a)**. Volume of mineralized tissue ingrowth into the area of interest **(b)** and bone to implant contact **(c)**. *p < 0.05; **p < 0.01; ***p < 0.005.

Four types of scaffolds were implanted in rabbits' calvarial defects. After 4 weeks, rabbits were killed and samples were harvested for analysis. We noticed that half of the Lattice group scaffolds occurred to be slightly broken. The results of bone ingrowth are given in Figure 4. At visualized 3D models of scaffolds, the blue color refers to the scaffold itself, and the pink color—to the mineralized tissue (Fig. 4a). The differences in the amount of bone tissue ingrowth between scaffolds were visible and were further quantitatively analyzed (Fig. 4b, c). Diamond-, Gyroid- and Lattice-based scaffolds induced more bone ingrowth than the Primitive-based scaffold. Statistically significantly more mineralized tissue was, however, found only in Diamond microarchitectures compared with Primitive microarchitectures (Fig. 4b).

Concerning bone-to-implant contact, Diamond and Gyroid yielded significantly higher values than Lattice but only Diamond was significantly higher compared with Primitive (Fig. 4c). For both measures, Diamond performs significantly better than Primitive but there is no significant superiority of Diamond over Gyroid.

## Discussion

TPMSs are promising microarchitectures to be used in bone substitutes because they combine natural bone characteristics as lightweight with high mechanical strength. In this study, we compared three different TPMS microarchitectures with a lattice microarchitecture based on mechanics, performance as a 3D-cell culture scaffold *in vitro*, and *in vivo* as a scaffold in a calvarial bone defect model.

Investigation of scaffolds based on the TPMS and Lattice designs by μCT scanning^38^ revealed the precision and the suitability of the 3D printing process for the production of ceramic scaffolds with TPMS microarchitectures. Scaffolds based on Primitive design had the highest volume, corresponding to the highest material consumption during the 3D printing process. Moreover, they were associated with the lowest porosity and surface-to-volume ratio among the manufactured scaffolds, which influences cell adhesion, proliferation, and differentiation.^39,40^

Analysis of compression tests for both Diamond and Gyroid designs revealed higher values of compression strength compared with Primitive and Lattice structures Besides, Diamond scaffolds exhibited the highest Young's modulus. Both measures are in line with results from polylactic acid–based scaffolds.^41^ The superiority of TPMS structures over the Lattice microarchitecture was shown earlier by others^42,43^ and could be explained by a more homogeneous stress distribution.^43^ It is worth mentioning that all tested scaffolds, both TPMS and Lattice, occurred to be in the range of natural bone elastic modulus.^37^ However, concerning the compression strength, only Diamond and Gyroid designs reached the corresponding values of bone tissue.^36^ Nevertheless, the relatively low elastic modulus of all tested scaffolds correlates with a lower chance of stress shielding after implantation.^44^ It is necessary to mention that the compression tests were conducted only unilaterally in the direction of the building layers and additional work would be needed to assess anisotropy in greater details.

Within our studied samples, different stress distributions were observed. In particular, during most compression tests only small pieces of Diamond and Gyroid samples were separated from the angles, whereas Lattice structures were always split by their layers, indicating a stress concentration at the connections of lattice struts.^10^ The breaking behavior affects the safety of scaffolds as in case of breaking into multiple pieces, the scaffold will lose its initial function, and additionally, the friction between these small parts and soft tissue may lead to wear particles and excessive damage.^45^ Therefore, preservation of the scaffold's integrity, as seen for Diamond and Gyroid, is preferable.

*In vitro* culture of hBMSCs showed that an increase in culture time led to a higher metabolic activity of hBMSCs as was shown in other studies,^46,47^ although, no difference between the control and osteogenic medium was observed. Furthermore, after 10 days, the scaffolds' pores were already colonized by cells (Fig. 2) and completely filled after 21 days of culture. In addition, some areas with dense extracellular matrix production and nuclei of mineralization were only observed in scaffolds cultured in an osteogenic medium and could be associated with an early ossification process.^48^

An increase of bone-related gene expression over time in hBMSCs cultured in the osteogenic medium compared with the control medium was revealed, which correlates with other studies,^49,50^ although no significant differences were found between the four scaffold types and 2D culture. This could be explained by the use of cells from five different human donors where the cellular composition is heterogenic and could impact the osteogenic potential as previously shown.^51^ The *in vitro* results let us assume that for wide-open porous 3D constructs in a static cell culture system, the microarchitecture in terms of surface characteristics, maximal fitting sphere (Table 3), or transparency (Table 4) does not affect survival, proliferation, and differentiation of hBMSCs.

To study the osteoconductive capability of our scaffolds, they were implanted into calvarial defects as previously described.^52^ After 4 weeks, half of the implanted Lattice samples appeared to be slightly broken in contrast to the TPMS scaffolds, which appeared to be still intact. Although the Lattice structure did not show the lowest mechanical characteristics in our study, these results correlate with the different breaking behavior of the scaffolds (Fig. 1). In contrast to the performed standardized unilateral compression tests, the direction of forces subjected to the scaffold *in vivo* is arbitrary, not standardized, and might lead to higher stress concentrations and failure at the connections of the struts.^53,54^ TPMS structures, however, are known to facilitate a more uniform stress distribution,^9,55^ which could lower the probability of scaffold failure to occur, irrespective of the direction of the force applied.

After μCT scanning of the samples after 4 weeks of implantation, bone ingrowth was quantified (Fig. 4). Concerning the bone volume, it was possible to observe a significant superiority of Diamond microarchitecture over Primitive. Among the reasons for this finding could be the highest value of the surface-to-volume ratio of the Diamond scaffold as well as its highest interconnectivity in comparison with other designs.^19^ Moreover, the low bone ingrowth in Primitive scaffolds could be related to the lowest porosity and surface-to-volume ratio of this microarchitecture among the studied designs.^33^

In previous work from our laboratory, we showed that the diameter of the largest 2D sphere fitting into the pore system of the scaffold has a strong influence on osteoconduction and bone regeneration, and should not exceed 1.53 mm in diameter.^33^ This number was not available at the beginning of this study. Despite the higher sphere diameter for the Diamond scaffold (1.17 ± 0.02 mm) in comparison with Gyroid and Lattice (0.81 ± 0.02 mm and 0.79 ± 0.02 mm, respectively), all these three microarchitectures were in the optimal range. However, the diameter of the largest 2D sphere for the Primitive microarchitecture-based sample was 1.63 ± 0.17 mm, which is beyond the recommended level.^33^ This could explain the lowest osteoconduction and bone ingrowth observed with this type of microarchitecture designed at a predefined minimal pore size of 0.8 mm.

Under the conditions tested, our data suggest that the microarchitecture of TPMS-based structures should not be defined only by the minimal diameter of the 2D sphere fitting into the scaffold of 0.8 mm but also by the maximal diameter, which should not exceed 1.53 mm. That these numbers could also hold for other materials like titanium was shown recently by us with a novel periodic minimal surface microarchitecture.^33^

Bone-to-implant contact is essential for the stability of an implant, correlates with high surface area,^56^ and is a measure of osseointegration.^57,58^ The Diamond microarchitecture compared with the Lattice and Primitive microarchitecture showed a significantly higher bone-to-implant contact (Fig. 4c). The lowest value for bone-to-implant contact was found in the Lattice microarchitecture, despite the fact that the surface area in the Primitive microarchitecture is lower than in Lattice. Therefore, microarchitecture in terms of the directionality of the building elements or transparency of the design plays a role in bone ingrowth patterns reflected by bone-to-implant contact.

The transparency of the Lattice microarchitecture is the highest of the tested designs (Table 4), and its straight elements compared with the curvy surfaces in the TPMS microarchitectures facilitate a straight ingrowth of newly formed bone between the struts with a low bone-to-implant contact ratio. That is in line with previous findings^7^ because bone formation in titanium and tricalcium phosphate lattice microarchitectures was observed predominantly between and not on the surface of struts, substantially minimizing the bone-to-implant contact. Thus, in wide open-porous scaffolds of high transparency the scaffold serves more as a guiding cue reflected by a low bone-to-implant contact ratio and less as direct support for bone ingrowth.

## Conclusion

In our study, we showed that fundamental differences in the algorithm of wide open-porous TPMS microarchitectures occurred to be a key factor for the osteoconductive potential *in vivo*. Over 4 weeks, only the Diamond-based scaffold showed a significantly higher bone ingrowth than Primitive structures and a significantly higher bone-to-implant contact ratio than Primitive and Lattice structures. For both measures, Gyroid microarchitectures did not perform significantly differently than Diamond. In combination with the good mechanical properties, low material usage, and therefore low cost of production, scaffolds based on the Diamond or Gyroid microarchitecture appear to represent promising bone substitutes for tissue engineering and regenerative approaches.

## Supplementary Material

Supplemental data
