# Supplementary material for: Triply Periodic Minimal Surface-Based Scaffolds for Bone Tissue Engineering: A Mechanical, In Vitro and In Vivo Study
Source: Tissue Eng Part A. 2023 Oct 11;29(19-20):507–17. doi: 10.1089/ten.tea.2023.0033 (PMC10611970; doi:10.1089/ten.tea.2023.0033)
Supplement: Supplemental data [file Suppl_FigS1.docx]

| 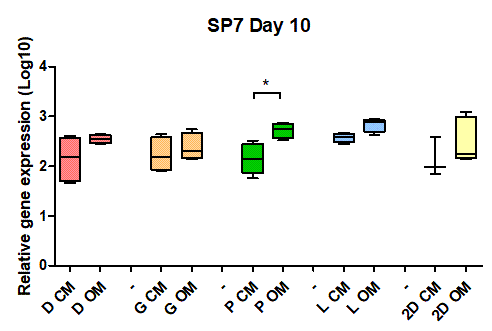 | 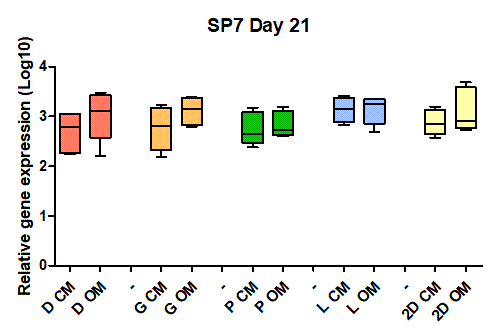 |
| --- | --- |
| 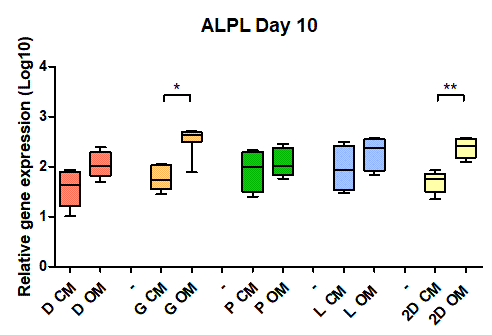 | 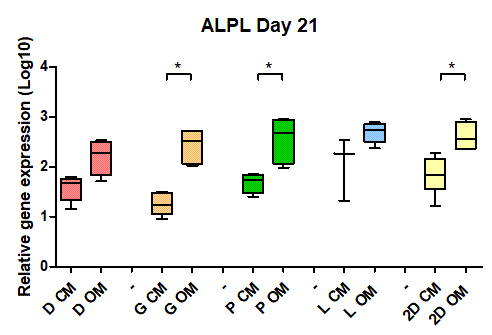 |
| 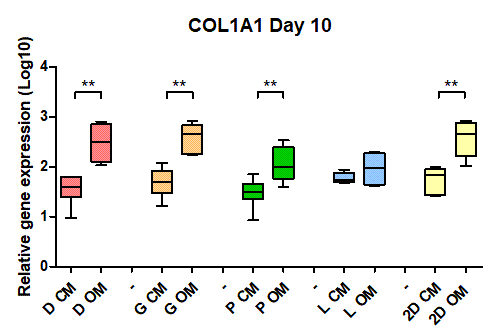 | 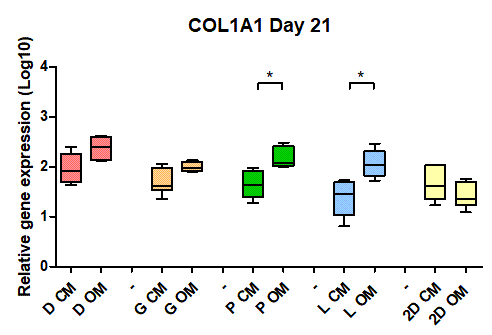 |
| 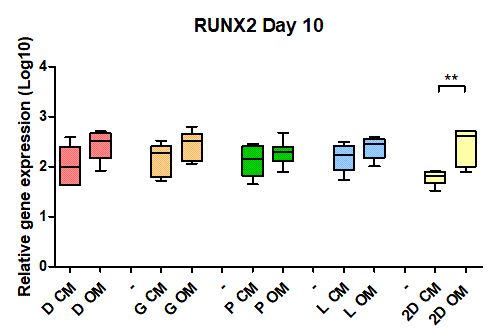 | 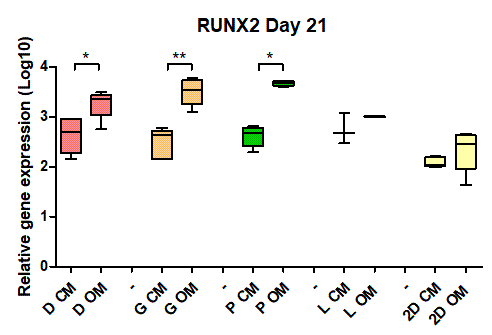 |
| 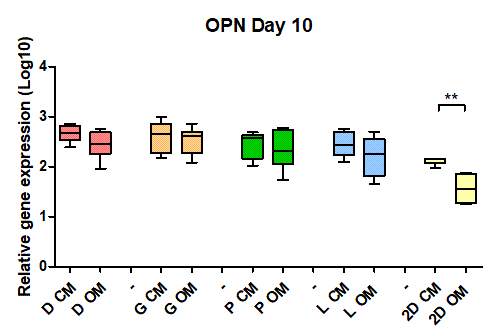 | 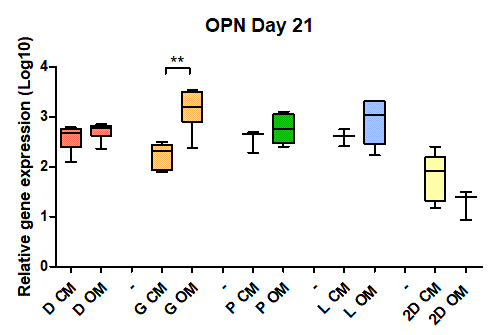 |
| 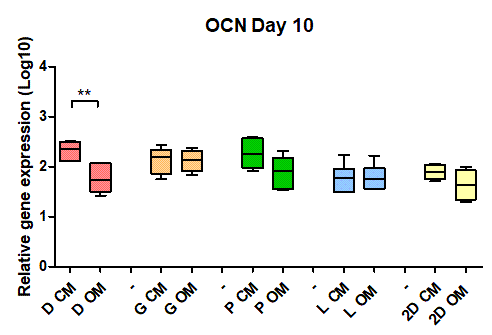 | 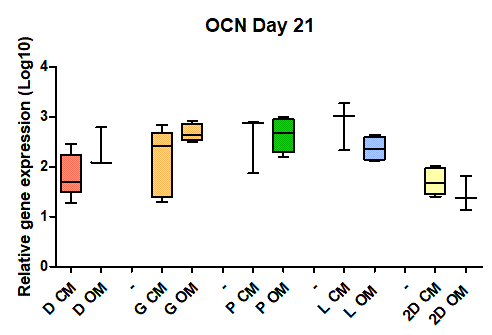 |
| 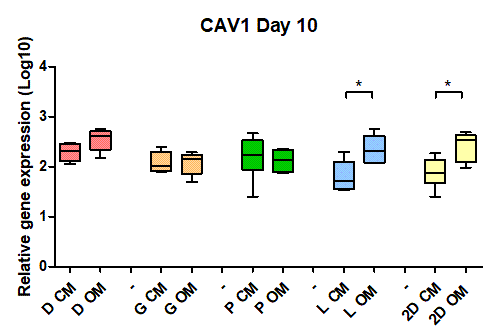 | 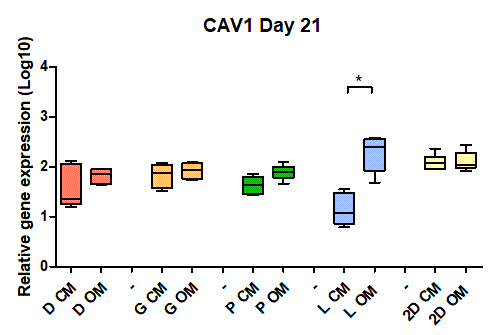 |

**S. 1:** Relative gene expression: D, G, P, L – Diamond, Gyroid, Primitive, Lattice microarchitectures; CM and OM – control and osteogenic medium. * p < 0.05; ** p < 0.01. Tested for Sp7 transcription factor (*SP7*), alkaline phosphatase (*ALPL*), collagen type I alpha 1 chain (*COL1A1*), *RUNX* Family Transcription Factor 2 (*RUNX2*), osteopontin (*OPN*), osteocalcin (*OCN*), caveolin 1 (*CAV1*).
